# Tuberculosis Elimination in the Asia-Pacific Region and the WHO Ethics Guidance

**DOI:** 10.3390/tropicalmed3040115

**Published:** 2018-10-31

**Authors:** Justin T. Denholm, Diego S. Silva, Erlina Burhan, Richard E. Chaisson

**Affiliations:** 1 Victorian Tuberculosis Program, Melbourne Health, and Department of Microbiology and Immunology, University of Melbourne at the Doherty Institute, Melbourne 3000, Australia; 2 Faculty of Health Sciences, Simon Fraser University, Vancouver, BC V6B 5K3, Canada; diego_silva@sfu.ca; 3 Department of Pulmonology and Respiratory Medicine, Faculty of Medicine Universitas of Indonesia/Persahabatan Hospital, Jakarta 13230, Indonesia; erlina_burhan@yahoo.com; 4 Johns Hopkins University Center for Tuberculosis Research, Baltimore, MD 21287, USA; rchaiss@jhmi.edu

**Keywords:** tuberculosis, ethics, World Health Organization, public health

## Abstract

The World Health Organization has produced ethical guidance on implementation of the End TB strategy, which must be considered in local context. The Asia-Pacific Region has important distinctive characteristics relevant to tuberculosis, and engagement with the ethical implications raised is essential. This paper highlights key ethical considerations for the tuberculosis elimination agenda in the Asia-Pacific Regions and suggests that further programmatic work is required to ensure such challenges are addressed in clinical and public health programs.

## 1. Introduction

In 2017, the World Health Organization (WHO) released *Ethics Guidance for the Implementation of the End TB Strategy* [1]. Ethical commentary frequently highlights key issues for consideration, with ongoing work required for contextualization in a variety of local settings. As part of engagement with questions of progressing towards TB elimination in the Asia-Pacific Region, then, it is also important to consider ethical emphases or challenges of particular importance. The purpose of this paper is to highlight key ethical considerations for the TB elimination agenda in the Asia-Pacific Region.

## 2. Latent Tuberculosis Infection

Expansion of latent TB infection (LTBI) treatment will form a key part of expanded programmatic interventions aimed at reducing TB incidence in the Asia-Pacific Region. As such interventions all involve potential harms and benefits for participants, appropriate selection of individuals and groups for testing and treatment is critical. We have written elsewhere about the ethical imperative to select people who are at significant-enough risk of future TB to warrant inclusion in such programs [2]. However, in the Asia-Pacific Region, there are also some specific ethical challenges with regard to ensuring that potential harms associated with these programs are minimized. There are two challenges we would highlight here for this region: The first is the selection of treatment regimens with lower risk of severe adverse effects, and the second is the, population risk factors that result in higher risk of severe adverse effects than in some other contexts.

With regard to treatment regimen, isoniazid monotherapy continues to be the most widely used treatment for LTBI in the Asia-Pacific Region. While strong randomized controlled trial data and programmatic experience elsewhere is available to support the use of several types of rifamycin-based short course therapy, such regimens have not been extensively adopted to date in the region [3,4]. Although there is likely to be a range of factors influencing programmatic approaches in this area, the higher unit cost of rifamycins and the lack of regulatory approval in some countries both present barriers to widespread use [5]. One additional limitation of some short-course regimens is the use of directly observed therapy, particularly in trials of weekly rifapentine and isoniazid (3HP), which increases the participant burden of LTBI treatment and is costly to programs. However, subsequent and ongoing studies have now also investigated 3HP using self-administered therapy and suggest equivalent adherence and completion in at least some settings [6,7]. The justification for directly observed treatment of active tuberculosis is largely based on preventing the emergence of drug resistance during treatment due to nonadherence. There is no documented evidence that treatment of latent tuberculosis selects for drug-resistant organisms, so supervision of preventive treatment lacks a strong public health rationale.

Where isoniazid has continued to be used, several population factors in the Asia-Pacific have also led to higher risk of serious adverse effects, particularly relating to hepatotoxicity. N-acetyltransferase (NAT) genotype, referred to as “acetylator status”, is linked to risks of toxicity, with slow acetylators having higher exposures to isoniazid and a greater risk of liver and other toxicities. The prevalence of slow acetylator status is both high and variable across the Asia-Pacific [8,9]. Many countries in the region also have high background rates of other comorbidities, such as hepatitis B infection and diabetes, which are linked to increased risk of severe adverse effects [10,11]. Assessment of acetylator status before starting LTBI therapy would allow better risk stratification, but such testing has not been promoted or adopted outside of research contexts. This is particularly challenging as TB programs have a duty to provide treatment as safely and effectively as possible. We would argue that investigating ways to increase awareness of acetylator status in clinically meaningful timeframes should be prioritized in this region, particularly where the ongoing use of isoniazid as the primary treatment for LTBI is planned.

## 3. Contact Assessment and Testing

Many countries within the Asia-Pacific Region have emphasized provision of LTBI treatment for only the groups at highest risk of progression, particularly children under the age of 5 and HIV-positive individuals who are contacts of smear-positive TB. While clearly appropriate, we would emphasize that a much larger group of people are at significant risk of TB disease and poor sequelae, and they should therefore also be included in efforts aimed at detection and treatment of LTBI. This includes adolescents who are increasingly recognized as critical in terms of both TB impact and transmission and contacts of multidrug-resistant (MDR) TB [12,13]. Although controversy persists regarding the optimal strategies for reducing risk after MDR TB exposure, such contacts are at high risk of developing the disease, and the best available data should be used to prioritize their wellbeing [14]. We would also highlight that while much of the emphasis on detection and treatment of LTBI is in low-incidence settings, LTBI treatment in high-incidence countries may provide significant benefit for affected individuals and should not be neglected when considering the best use of programmatic resources in specific contexts [15].

One additional issue related to LTBI in the Asia-Pacific arises from recent changes to WHO guidance on contact assessment, including the statement that certain groups of household contacts (children aged less than 5 and HIV-positive individuals) can be treated with preventive therapy in the absence of a test for LTBI [16]. Questions have been raised as to whether it is ethical to offer preventive therapy in the absence of a test, with some arguing that it is wrong to expose individuals who would test negative to the risks of treatment without the corresponding benefit. However, we would argue that, ethically speaking, what is most important is to offer therapy to individuals who have an appropriate risk of developing TB and therefore stand to benefit from treatment. Such a risk assessment is substantially informed by contact identification; these individuals with household exposure who have important personal risk factors for progression to active disease are at sufficient risk to justify treatment and, in fact, to obligate offering treatment even in situations where a test for LTBI cannot be conducted. While testing for LTBI remains important for further refining risk estimates and promoting individual autonomy, the lack of test availability should not be used to justify a failure to provide a life-saving intervention for these vulnerable groups. In addition, in high TB transmission settings, HIV-infected individuals with negative tests for LTBI have been shown to benefit from preventive therapy to the same extent as those with positive tests [17].

## 4. Migration

International migration is recognized as one of the central drivers of development in the Asia-Pacific Region, with an estimated 59.3 million migrants in 2015 [18]. Much of the approach to migration and the TB elimination agenda has concentrated on permanent migration, particularly from high- to low-incidence settings. Migration in this pattern provides important opportunities for intervention and long-term health promotion, such as LTBI testing and treatment, which reflects the changing risk of TB exposure experienced. However, permanent relocation to low-incidence settings is only a small proportion of migration in the Asia-Pacific Region, with a considerable majority of migrations occurring between high-incidence settings and frequently on a temporary basis, particularly for occupational purposes [19,20].

It is unethical for employers to relocate employees into settings where they do not have appropriate access to free and timely healthcare. In principle, we would argue that the provision of genuine universal healthcare services should allow for complete access to care for all residents, regardless of migration status. In practical terms, however, temporary migrants in many countries may not have such full access, and responsible employment of migrants should ensure that individuals are not at risk of poor or delayed healthcare due to their location. This ethical obligation will generally mean that employers must provide health insurance as part of employment contracts. Our experience is that, in practice, even where such health coverage is available, it is often inadequate, thus leaving sick individuals potentially liable for considerable costs. Existing arrangements also rarely adequately consider the impact of lost wages and family impact from a condition that might require many months of treatment, and we would suggest that there is an ethical imperative for employers to ensure that holistic care provisions are made available for international workers.

## 5. Catastrophic Costs and Financial Burden

While much of the emphasis in TB elimination strategy highlights the targets and pathways for incidence reduction, it is critical to reinforce the equally central aim of eliminating catastrophic costs caused by TB [21]. Across the Asia-Pacific, a wide variety of healthcare and social support systems are in operation, including substantial involvement of the private sector in many areas. Clearly, there is a need to ensure that TB treatment itself is free and accessible, including treatment for LTBI. While this is an important baseline requirement—and one where there are still gaps in comprehensive programmatic support in many parts of the region—provision of free TB treatment itself will not address the issue of catastrophic costs. The most significant costs associated with TB frequently come through lost opportunities for employment or other occupational activities, caring for family members, interrupted education, and other ways in which serious and prolonged health conditions like TB can impact individuals and families. Therefore, a much broader, wholesale reform of welfare and social support services is required, including universal healthcare to ensure that more than just TB drugs are provided without risk of catastrophic cost. Commitment to the TB elimination agenda means an ethical obligation for governments to work toward providing a complete range of personal and social support services in order to ensure that individuals and families affected by TB do not suffer catastrophic costs. The varying structures for provision of essential healthcare in countries of the Asia-Pacific will require governments and TB programs to work with a range of stakeholders across both the public and private sectors and a commitment to ensuring that the elimination of catastrophic costs is highly prioritized over other stakeholder interests.

In considering the financial burdens of TB across the Asia-Pacific, we would also highlight the importance of recognizing the fact that such costs are not distributed equitably and may disproportionately impact individuals and groups who are already vulnerable. This includes those marginalized for reasons of race, gender, sexuality, or the presence of other stigmatizing conditions. Consideration must be given to such factors in local contexts, and programmatic interventions should actively consider how such inequities can be prioritized and redressed.

## 6. Healthcare Workers

Healthcare workers in many settings are recognized to be at increased risk of both TB infection and disease, including in the Asia-Pacific Region [22]. The TB elimination agenda requires the dedicated and persistent work of healthcare professionals, and the risk they face of infection in doing so creates important ethical obligations on the healthcare systems in which they work. These obligations firstly mean the responsibility to provide a safe workplace, including infection control policies and practices to limit risk, and provision of appropriate and accessible personal protective equipment. It should also include rational and nondiscriminatory approaches to LTBI testing and treatment aimed at limiting the consequences if infection was to occur. While we would argue that these responsibilities lie with healthcare systems, individual healthcare workers may also perceive that they have professional or altruistic obligations to work despite such risks. An exploration of these obligations is beyond the scope of this present work, but we would point out that any such responsibilities are not unlimited, and healthcare workers may be justified in considering action (as individuals or collectively) to avoid risk where appropriate healthcare system obligations are not met.

## 7. Conclusions

The Asia-Pacific Region is a diverse context and presents a variety of challenges to TB elimination. While distinctive epidemiological issues are generally well recognized, we have highlighted here that there is also variation in ethical emphases that should be considered as an essential part in working towards TB elimination in the region.
